# Exploring the public’s perception and understanding of Parkinson’s disease in Ireland: a study protocol

**DOI:** 10.1186/s12877-025-06091-5

**Published:** 2025-07-02

**Authors:** Sophie Crooks, Gary Mitchell, Lisa Wynne, Gillian Carter

**Affiliations:** 1 School of Nursing and Midwifery, Queen’s University Belfast, Belfast, Northern Ireland UK; 2 Parkinson’s Ireland, Dublin, Ireland

**Keywords:** Parkinson's disease, Stigma, Lived Experiences, Parkinson's Stigma, Quality of Life, Systematic review

## Abstract

**Background:**

There appears to be a lack of empirical investigation on how the public currently understands Parkinson's Disease (PD)and the impact this has on people with PD and their carers, across Ireland. This research hopes to build a greater public understanding of PD, reduce stigma and improve the quality of life for people with PD. The aim of this study is to (1) explore public perceptions, awareness and understanding about PD across the island of Ireland and (2) discover how this impacts people with PD and their families.

**Methods:**

This study will adopt a mixed-methods, sequential explanatory methodology across four phases. This will include a systematic review of the literature, semi-structured interviews with people with PD and their carers, and focus groups with specialist PD professionals to determine how public perception impacts those living with PD. Following this, an online Delphi survey will be conducted with PD experts to determine a list of key priority areas for PD education.

**Discussion:**

PD affects a large population of people worldwide and this number is expected to increase due to a progressively ageing population. There is a lack of empirical research exploring what the public know about PD and the impact this has on people living with the disease particularly in Ireland, therefore it is vital that we determine a list of priority areas for increasing PD awareness to improve the quality of life of those living with PD.

**Supplementary Information:**

The online version contains supplementary material available at 10.1186/s12877-025-06091-5.

## Introduction

Parkinson’s Disease (PD) is the fastest growing neurological condition in the world and is the second most common after dementia [1, 2]. It is estimated that there are over 8.5 million people living with PD worldwide, with approximately 145,000 of those living within the UK [3, 4]. It is expected that by 2025 the prevalence of PD will increase by 23.2% because of population growth and an increasingly ageing population [5]. The exact cause of PD is unknown, however, there are several risk factors that increase the likelihood of developing the disease including age [6], gender (male) [4], and some environmental factors such as pesticide exposure and rural living [7, 8].

There is currently no cure for PD, however, there are strategies such as medication, physiotherapy, occupational therapy, and surgical intervention, which can help those with PD manage symptoms and live with the disease. Due to its highly individualised and complex nature, PD requires interprofessional support from multiple disciplines including nurses, doctors, and physiotherapists [9]. Experts specialising in PD care have specialist experience, knowledge and skills and work closely with people living with PD and their families, helping with medication management, offering advice and information, and providing emotional support.

Scientific understanding of the physical symptoms of PD is now well established, therefore focus must shift to the impact of the disease and challenges people with PD face day-to-day [10]. Recent research exploring the psychosocial impact of PD has positively associated faster PD progression and worsening symptoms with depression, anxiety, and fatigue [11]. These psychosocial difficulties, alongside physical challenges, can be detrimental for people with PD by decreasing their involvement in social activities, therefore reducing social contact, and resulting in social isolation [12]. Furthermore, there also appears to be a lack of empirical investigation on how the public currently understands PD, particularly in Ireland. Findings from a recent scoping review suggest public knowledge and awareness of PD is poor [13]. This review included global research and highlighted a particular lack of knowledge alongside conflicting views of signs, symptoms, causes and treatment of PD. It also identified public perceptions about quality of life in PD, with many studies associating PD with social isolation, depression, and loss of independence [14–18]. In the review, education was identified as a critical step to improve knowledge and raise awareness of PD, including electronic learning modules, use of social media and building age-friendly communities [19, 20]. Implementation of these resources aim to improve awareness, build community support, and provide a sense of empowerment for those living with PD. This is critical as without good awareness and knowledge of PD, the public are less likely to be able to effectively support people with PD in their local communities and plan or deliver meaningful social interventions.

In addition to how the public understands PD, there is also limited research exploring how people with PD experience life in their own communities and the role that the public play [11, 21, 22, 23]. Participants in one study by Nazzal and Khalil [24] believed people in the community perceive individuals with PD differently, which they felt could be caused by a lack of public understanding of the disease. Gaining an insight into the lived experiences of people with PD in their communities is essential for building an understanding, reducing stigma and for improving their quality of life.

Exploring the experiences of people with PD and their carers first hand is essential however, exploring different perspectives of the experiences of people with PD is helpful for better understanding of how they live in their communities. Given their degree of specialisation and close involvement with their patients, professionals who specialise in PD are likely to have a good knowledge of how people with PD experience life in their communities [25, 26]. Despite this, there is lack of research about the knowledge these professionals have of the experiences of people with PD and the role they play in helping people with PD live better within their communities. This could help inform future research and development of educational resources that help increase understanding of the disease, as well supporting those with PD to live better within their communities.

## Methods/design

### Methods

**Fig. 1:**
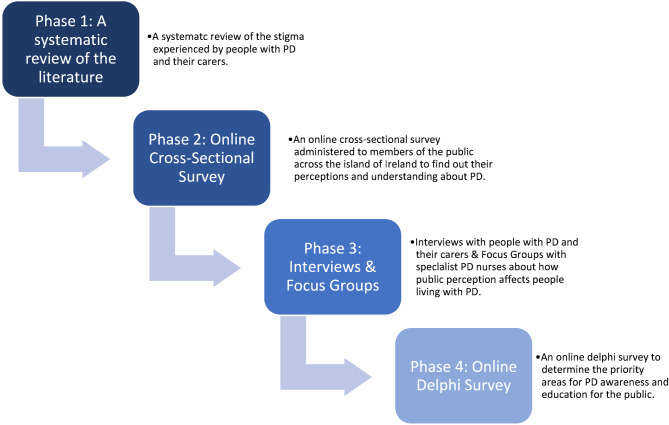
Study Overview

This study will use a mixed methods design across four phases (Fig. 1) to explore the public perceptions and understanding about PD in Ireland. Mixed-methods research combines elements of both qualitative and quantitative approaches, allowing researchers to explore a diverse perspective and gain a deeper understanding of a phenomenon [27–29]. Mixed methods are useful for gaining a better understanding of relationships between qualitative and quantitative data by providing participants with the opportunity to share their experiences and elaborate on their responses, further enriching the evidence [30].

Specifically, this study will adopt a sequential explanatory mixed methods design. This method is characterised by an initial quantitative phase, which will be the primary method, followed by a qualitative data collection phase. An initial quantitative cross-sectional survey will gain an insight into the public perceptions and understanding about PD, providing a general understanding of the research problem [31]. Then the qualitative data will incorporate semi-structured interviews with people living with PD and their carers, and focus groups with specialist PD professionals to develop an understanding of the experiences of those living with PD within their communities. This data will provide a more refined and in depth understanding of the statistical results [32]. Following this, a Delphi survey will be conducted with a panel of PD experts to determine priority areas for public education and awareness for PD.

### Phase one: a systematic review of the literature

Phase one will involve a mixed methods systematic review of the literature to synthesise current evidence on the stigma experienced by people with PD and their carers [PROSPERO - CRD42023399343].

### Review question

What is the stigma experienced by people affected by Parkinson’s disease from the public?

### Aims and objectives

The study aims to explore the prevalence and impact of PD stigma across diverse social and cultural contexts while investigating its physical, psychological, and social ramifications on individuals with PD, caregivers, and families. It seeks to uncover the roots of PD stigma, including misconceptions and stereotypes, and identify research gaps. Through a systematic review, it aims to synthesise existing literature, discern prevalent themes, and offer evidence-based recommendations for future research and stigma reduction interventions. Additionally, the study aspires to foster public awareness and empathy toward PD-related stigma, promoting societal understanding and support for affected individuals.

The findings of this review will help inform phase two, three and four of the study by identifying areas of importance to be explored further. This review will follow guidance from Joanna Briggs Institute (JBI) Systematic Reviews [33]. Systematic reviews are well defined and accepted internationally, with a number of key defining features including: well determined eligibility criteria, objectives and research question, quality appraisal of included studies, comprehensive search, clearly reported methodology, analysis of data extracted from the research and synthesis and presentation of the findings [34].

### Search strategy

A search of the following databases will be conducted: CINAHL, MEDLINE, EMBASE, PsycINFO, Web of Science and Cochrane Library. These databases were chosen as they are strong sources of health evidence for nursing and allied health literature. Search terms will be created based on the three main themes of Parkinson’s disease, stigma (and related terms) and public perception.

Eligibility criteria will be clearly defined through inclusion and exclusion criteria. Eligible studies will be those exploring the experiences of people with PD and their carers within their community. Studies must involve people diagnosed with PD and those providing informal care. The systematic review software Covidence will be used for the removal of duplicates, screening, and data extraction. The Joanna Briggs Institute (JBI) data extraction tool will be used to extract data relevant to this review.

### Phase 2– online cross-sectional survey

An online cross-sectional survey will be developed and administered to members of the public across the island of Ireland to determine the public perceptions and understanding of PD. The survey will be created electronically on Microsoft (MS) Forms and made available online to reach a large population. The content of two previously validated questionnaires (Youn et al. [35]; The Cervical-Cancer-Knowledge-Prevention-64 (CCKP-64) questionnaire [36] will be combined and modified to create the survey for this study which will suit the population and objectives. Despite the CCKP-64 questionnaire not relating to PD, aspects of the content were deemed appropriate to modify for the questionnaire for this study. The online survey will be piloted with approximately 10 people using the same process. Members of the public who are over the age of 18 and live in the Northern Ireland (NI) or the Republic of Ireland (ROI) will be the participants for this phase of the study.

### Recruitment for online cross-sectional survey

Participants will be recruited using convenience sampling via social media platforms including Facebook, Instagram, and X, which will be utilised to disseminate the survey to the wider population. A link to the survey will also be advertised on the social media pages of Parkinson’s United Kingdom (UK) Northern Ireland (NI), Parkinson’s Ireland, Age Northern Ireland, and Age Friendly Ireland. To ensure comprehensive coverage of public perceptions of PD, this study aims to recruit approximately 300 participants, aligning with the sample sizes observed in recent scoping reviews [13]. This range allows for a diverse representation of perspectives and ensures thorough exploration of the research topic. A list of survey questions that will be used in the survey can be seen in Additional File 1.

### Phase 3– semi-structured interviews and focus groups

This phase will be conducted in two stages:

Stage 1: Semi-structured interviews with people with PD and their carers.

Stage 2: Focus Groups with professionals who specialise in PD care.

### Stage 1: semi-structured interviews with people with PD and their carers

A sample of people living with PD and their carers will be invited to take part in semi-structured qualitative interviews, they will be conducted to investigate how public perception and understanding affects people living with the condition. Interviews will involve people with PD on their own or dyad interviews with people with PD and their caregivers. Interviews will take place either online, telephone or face-to-face, depending on participant preference. Online interviews will take place using the MS Teams platform due to its ease of use, convenience for both participant and researcher and ability to video and audio record sessions [36]. Face-to-face interviews will take place in a private location at the convenience of the participant, such as the participants private home if they are comfortable. The location should allow the participant to feel safe and speak privately and freely without interruption [37]. Interviews will be conducted at dates and times convenient to the participant. With permission of the participants, all face-to-face and telephone interviews will be audio-recorded. An interview guide will be developed based on findings from the systematic review and the key findings of the online survey, that determine priority areas of interest for people living with PD in their community. Interviews will continue to be conducted until data saturation is reached and the researcher determines no additional information will be attained. A proposed interview guide can be seen in Additional File 2.

### Recruitment for semi-structured interviews

Participants will be recruited using convenience sampling via gatekeepers within Parkinson’s Ireland and Parkinson’s UK NI. Participants must be a person living with PD, carer or family member of someone with PD, over the age of 18 and able to communicate in English. The recruitment phase will be open to all individuals, regardless of their ethnic or cultural background. Gatekeepers will contact potential participants via email or in-person during support group meetings and provide information about the study, including contact details of the researcher. Emails will attach a Participant Invitation Letter and Expression of Interest Form and Participant Information Sheet, providing further details about the study. Potential participants attending in-person support group meetings will be given the same information in a hard copy. Following expression of interest from potential participants, the researcher will set up a virtual meeting (via MS teams) or telephone call with the participant and their family member/carer if appropriate. This initial meeting will provide clarity to the participant on what the study entails and will provide the opportunity to answer any questions, alongside building researcher and participant rapport. It is anticipated that 10–12 interviews will take place, as Hennink & Kaiser [38] suggest 9–17 interviews is sufficient for reaching data saturation in qualitative research.

### Stage 2: focus groups with professionals

A sample of professionals that provide specialist care to people with PD and who in either NI or ROI will be invited to take part in one focus group interview to investigate how public perception and understanding affects people living with PD. These professionals are likely to have a good knowledge of how people with PD experience life in their communities given their close involvement with people living with PD and degree of specialisation. Focus groups will take place online or face-to-face. Due to the heavy workload and varied schedules of HCPs, in-person qualitative data collection can be challenging [39] therefore the option of an online focus group interview will be given. Online focus groups will enable wide geographical reach across the island of Ireland, removing the requirement to travel to a location. The focus group with professionals in ROI will take place online due to geographical location and travel requirements. Professionals within NI will be offered the opportunity to attend the focus group in-person, however this is not a requirement, and an online session will be facilitated if it is more suitable for participants.

### Recruitment for focus groups

Professionals specialising in PD care will be recruited via purposive sampling using gatekeepers from Parkinson’s UK NI and Parkinson’s Ireland. Gatekeepers will contact potential participants via email or through networking events. The email will have attachments containing Participant Invitation Letter, Expression of Interest Form and Participant Information Sheet. Potential participants attending in-person meetings or networking events will be given the same information in hard copy. The researcher will contact those interested in taking part via email to obtain online written consent and provide the opportunity to clarify any queries before scheduling the focus group interviews. Two focus groups will take place, one in NI and one in ROI. The aim will be to conduct focus groups comprising of approximately eight participants in each.

### Phase 4- Delphi survey

A Delphi survey will be conducted with guidance from the findings of the systematic review, cross-sectional survey, interviews and focus groups to determine what PD experts perceive to be the priority areas for PD education and awareness. Delphi survey methods are useful for obtaining a consensus to formulate recommendations for action and key priority areas [40]. This is a systematic, rigorous method involving group facilitation techniques with multiple stages, designed to transform opinion into a group consensus (Hasson et al., 2000). Experts will be recruited for this Delphi phase, which are those who possess knowledge in a particular area and are willing to share their opinions. In this study, experts would be those living with PD, those who are caregivers for those with PD, healthcare professionals (HCPs) who work with people with PD such as specialist PD nurses, neurologists working with PD, PD advocates and charity members. PD experts will be recruited through gatekeepers within Parkinson’s Ireland and Parkinson’s UK NI post interview phase.

Empirical findings from phases one to three will be used to comprise the initial Delphi items. The conventional Delphi survey design has four rounds, however, it is anticipated that three rounds of surveys will be sufficient to reflect on group responses and achieve consensus amongst PD experts across Ireland. The previously conducted scoping review [13] and findings from phases one to three will provide an adequate literature base to allow for three Delphi rounds to determine priority areas for PD awareness [40, 41]. Following the final round of survey, an online meeting with participants will be held to determine a consensus on the perceived priority areas for PD awareness. Determining consensus in a Delphi survey is open to interpretation, however Miller [42] explains how consensus on a topic can be determined if a certain percentage of votes falls within a prescribed range. The recommended percentage to determine consensus varies between the literature. We aim to achieve a consensus of 75% for this study as we are aiming to provide streamlined education priorities for policy, practice and third sector organisations. The Delphi survey will take place online due to the convenience for participants and data management advantages [43].

### Recruitment for Delphi survey

This stage of the Delphi process involves recruitment of informed individuals who have knowledge of the topic being investigated [44]. Selection of experts for this study will include people with PD and their carers, healthcare professionals such as PD specialist nurses, doctors and pharmacists, advocacy groups and charities. PD experts will be recruited through gatekeepers within Parkinson’s Ireland and Parkinson’s UK NI post interview phase. The researcher will contact those who have consented to take part in the Delphi survey via email with links to the survey. Those who self-nominate during the interview phase (people with PD/carers) and express an interest in taking part in this, will be recruited for the Delphi survey phase. Samples within a Delphi survey tend to focus on quality of participants in relation to their expertise and knowledge in the specific area of interest as opposed to number of participants. However, a recommended number of 12 participants is considered appropriate to enable consensus to be achieved [41, 45], therefore we aim to recruit 12–15 participants for this stage.

### Data analysis

#### Quantitative data

Data from cross-sectional surveys in phase three will be collected and transferred from MS Forms to be analysed using Statistical Package for Social Sciences (SPSS) software version 27 [46]. Descriptive statistics will offer insights into participants knowledge levels and perceptions, while chi-square tests will explore associations such as the link between knowledge levels and treatment awareness. Subgroup analyses by demographics such as age, gender and education will offer insights into variations within the public’s perceptions and knowledge.

Descriptive statistics will be used to report on results of the Delphi survey. Initially, a questionnaire will be created and disseminated to a panel of experts to determine key areas of importance within the topic [44]. Participants will be asked to rank several statements, using the Likert scale as stated previously (Sect. 4). The surveys collected via MS Forms will be downloaded onto a password-protected laptop and transformed into a tailored Microsoft Excel spreadsheet. This spreadsheet will then be exported to IBM SPSS^®^ Statistics Version 27 [46] as needed for conducting descriptive data analysis, including percentage calculations, median values, and interquartile ranges (IQR). This analysis will effectively present the quantitative data, particularly focusing on the Likert scale responses for each statement. Upon receiving completed surveys, responses will be assigned numerical values according to their perceived importance: Highest Importance = 5, High Importance = 4, Moderate Importance = 3, Low Importance = 2, and Very Low Importance = 1. Percentage calculations will be performed to determine the level of agreement for each statement. Given that Likert scales capture ordinal data, the median Likert scale response and interquartile range (IQR) will be computed for each statement. Statements with a median value of 4 or 5 (indicating high or highest importance) and a 25th percentile (P25) of ≥ 4 will proceed to the next stage. Essentially, only statements with at least 75% of participants identifying them as high or highest importance will be considered for inclusion in subsequent phases. Statements meeting the criteria of a median value of 4 or 5 but having a P25 of < 4 will be retained for the next round of the online survey.

After the completion of the final online survey round, inclusion in the research action plan will be limited to statements with a median value of 4 or 5 and a 25th percentile (P25) of ≥ 4. Criteria that do not achieve consensus will be preserved for future exploration. The expectation is that a progressive reduction of survey statements from 30 to 15 over three Delphi rounds will facilitate the attainment of consensus among participants.

#### Qualitative data

The one-to-one interviews and focus groups taking place will be video and audio recorded to capture the words of participants. This allows the researcher to concentrate and listen to the participant, without the added distraction of writing notes. Interviews and focus groups will be conducted by one member of the research team. Following this, interviews and focus groups will be transcribed verbatim [47]. During this process, the researcher will use a non-identifying variable (such as a number) to identify each participant and will remove any other identifying variables such as name, workplace, or place of birth to ensure anonymity and protect participant confidentiality [47]. Transcriptions of recordings will be uploaded for analysis using NVivo 11 management software. Qualitative data will be analysed using thematic analysis. This method is useful for identifying, analysing, and reporting patterns in data, helping the researcher understand experiences, thoughts, or behaviours across data [48–50]. This method allows themes to be generated and involves a six-step process as recommended by Braun and Clarke [49]: (1) Familiarising yourself with the data, (2) generating initial codes, (3) searching for themes, (4) reviewing themes, (5) defining and naming themes and (6) producing the report/manuscript.

### Consent process

Consent to participate in this study will be obtained from all participants (members of the public, people with PD/carers, healthcare professionals/experts in PD). Participants will be aware that involvement in the study is entirely voluntary and that they can withdraw from the study at any time without justification. The research team will acquire written consent online via MS Forms or email. If unable to provide written consent, consent will be obtained verbally through audio recording on MS teams or via telephone call. As phase three involves interviews with people living with PD, capacity will be assessed prior to involvement and all participants must be deemed to have capacity to take part in the study. Gatekeepers within Parkinson’s Ireland and Parkinson’s UK NI will determine the capacity of potential participants with guidance from the Mental Capacity Act (2016) (health-ni.gov.uk, 2024) in NI and Assisted Decision-Making (Capacity) Act (2015) (citizensinformation.ie, 2024) in ROI. These organisations have agreed to take responsibility for ensuring possible participants have the capacity to provide consent to take part in the study [51, 52].

### Ethics and governance

This study has received ethical approval from the Faculty of Medicine and Health Sciences, Queen’s University Belfast (MHLS23_132). This study will not take place in a clinical setting, therefore does not require NHS ethical approval or specific hospital trust approval. This study will be conducted in accordance with the Declaration of Helsinki. The researcher will obtain consent from all participants for online interviews to be recorded in accordance with General Data Protection Regulation (1995) (Ireland) and Data Protection Act (2018) (NI) and data will be stored securely on a private computer. Face-to-face interviews will take place in a private location at the convenience of the participant. With permission of the participants, all face-to-face and telephone interviews will be audio-recorded. Interviews taking place on MS Teams will be video recorded. Recordings will be downloaded to the secure network in Queen’s University Belfast. All recordings will be erased from the secure network after transcription. Informed consent will be collected from all participants involved throughout the study, with involvement kept confidential. Participant information will only be available to members of the research team. The identity of participants will be anonymised for all related publications and study outputs.

### Rigour

This study employs a mixed methods approach, therefore rigour is assessed in different ways. In quantitative data analysis, rigour entails ensuring validity and reliability by accurately measuring intended constructs and maintaining consistency in measurement conditions [53, 54]. For qualitative data derived from semi-structured interviews and focus groups, Lincoln and Guba’s (1985) four-step criteria will guide rigor: credibility, transferability, dependability, and confirmability [55]. Credibility will be upheld through member checking, where participants validate research findings via interview transcripts. Transferability will be established by detailing participant characteristics and research methods for potential applicability to other contexts. Dependability will be achieved by consistent research approaches documented for external critique, including clear records of research meetings. Confirmability will ensure research findings remain unbiased and respondent-driven, which will be facilitated by meticulous record-keeping throughout the study. These rigorous approaches collectively will enhance the trustworthiness and reliability of both quantitative and qualitative findings.

### Dissemination

It is envisaged that study outputs will be disseminated widely through several methods, including open-access articles in academic journals, scientific conference presentations, webinars, and on social media platforms (e.g., X and Facebook).

## Discussion

This study outlines the research approach for exploring the public perceptions and understanding about PD. Educating the public about PD is vital for increasing awareness, promoting early detection, and reducing stigma. By raising awareness of the signs and symptoms, individuals are empowered to seek timely diagnosis and access support services, improving their quality of life. Additionally, public education initiatives will help dispel misconceptions, foster understanding, and drive research efforts for better treatment. By promoting accurate information and advocacy, public education plays a crucial role in supporting individuals living with PD and their families and helping them live better within their communities.

While global trends provide a useful benchmark, we anticipate that public perceptions and awareness of PD in Ireland may differ in meaningful ways due to sociocultural, healthcare, and media-related factors unique to the Irish context. This study seeks to uncover these context-specific perspectives, contributing to a deeper understanding of PD awareness in Ireland. The findings will inform the development of targeted and culturally appropriate public health strategies to support those affected by PD.

## Electronic supplementary material

Below is the link to the electronic supplementary material.

Supplementary Material 1: Additional File 1 Online cross-sectional questionnaire.

Supplementary Material 2: Additional File 2 Proposed Interview Guide.

## Data Availability

No datasets were generated or analysed during the current study.
