# Supplementary material for: Exploring the public’s perception and understanding of Parkinson’s disease in Ireland: a study protocol
Source: BMC Geriatr. 2025 Jul 2;25:441. doi: 10.1186/s12877-025-06091-5 (PMC12220121; doi:10.1186/s12877-025-06091-5)
Supplement: Supplementary file 2 — Supplementary Material 2: Additional File 2 Proposed Interview Guide. [file 12877_2025_6091_MOESM2_ESM.docx]

**Additional File 2 – Proposed Interview Guide**

A sample of people living with PD and their carers will be invited to take part in semi-structured qualitative interviews which will be conducted to investigate how public perception and understanding affects people living with the condition.

We are interested in exploring the experiences of people living with Parkinson’s disease and their carers on the public experience living with PD.

**Introduction:**

1. How long have you been living with Parkinson’s disease?

**Understanding Experiences:**

1. Can you describe your typical experiences when you're out in public places? How does Parkinson's disease influence these experiences?
2. What challenges do you encounter in public settings due to your Parkinson's symptoms? Can you give specific examples?
3. How do you feel about interacting with others in public while dealing with Parkinson's symptoms? Are there any situations that make you feel more self-conscious or uncomfortable?
4. Have you noticed any differences in how people react or respond to you in public since your Parkinson's diagnosis? Could you share a specific situation that stood out to you?
5. Are there particular places or situations in public where you feel more at ease or less affected by your Parkinson's symptoms? What makes these situations different?
6. How do you manage your Parkinson's symptoms when you're in public? Are there strategies or techniques you use to cope with challenges you face?
7. Are there any changes you've made in your daily routine or activities due to the challenges you experience in public? How have these changes impacted your life?
8. Have you ever encountered any misconceptions or misunderstandings about Parkinson's from the public or others you've interacted with? How did you handle such situations?

**Support and Services:**

1. How do you think public spaces and environments could be more accommodating or supportive for individuals with Parkinson's disease?
2. What kinds of support and services do you think people with Parkinson's need to enhance their quality of life in the community?
3. In your experience, are there any gaps or areas where improvements could be made in terms of community-based support for individuals with Parkinson's?

**Communication and Awareness:**

1. How can we work together (researchers, healthcare professionals, people with PD) with the community to improve understanding of Parkinson's disease?

**Social and Psychological Impact:**

1. Can you tell me about a positive experience you have had in your community since being diagnosed with Parkinson’s disease?
2. Can you tell me about a negative experience you have had in your community since being diagnosed with Parkinson’s disease?

**Barriers to Engagement:**

1. Are there factors that might prevent you or other people with Parkinson's from actively participating in community activities or accessing services? How can these barriers be overcome?

**Future Improvements:**

1. In your view, what steps could be taken to create more inclusive and supportive communities for people with Parkinson's?
2. What changes or initiatives do you think would have the most significant impact on improving the lives of individuals with Parkinson's in their local areas?

**Conclusion:**

1. Is there anything else you would like to share about your experiences and perspective on living with Parkinson's in your communities?
